# The approach taken to reducing the risk of transfusion related acute lung injury in Canada

**DOI:** 10.4103/0973-6247.42696

**Published:** 2008-07

**Authors:** G. H. Growe, T. R. Petraszko, Mark Bigham

**Affiliations:** *Canadian Blood Services, BC and Yukon Centre, Vancouver, BC, Canada*

**Keywords:** Transfusion related acute lung injury risk reduction

## Abstract

Transfusion related acute lung injury (TRALI) has become a major reported cause of severe transfusion reactions and mortality. Over the past four years significant changes have been taken in Canada in order both to improve the recognition of the risk and to decrease its incidence. An international meeting was held in April of 2004 entitled “Towards an Understanding of TRALI". As a result of the analysis and recommendations from this meeting, the Canadian Blood Services established an ongoing review committee and established a laboratory diagnostic facility to identify at risk donors and recipients. A system has been developed to identify implicated donors and exclude them from the blood donor pool. Other steps have been taken to exclude potentially high risk donors, such as previously pregnant females, from the plasma and platelet donor pool. A considerable amount of education also has been offered to clinical services in the country. This paper summarizes the definitions, categorizations of implicated donors, and the ongoing precautionary activities related to plasma products. Noted within the article are the methods used for locating and selecting data. These were primarily based on the international TRALI conference in 2004, and from ongoing discussions and information provided by the Canadian Blood Services TRALI Review Committee. No ethics referral or approval was requested, and a summary is included in the article.

## Introduction

Transfusion related acute lung injury (TRALI) is a major cause of severe transfusion reactions and mortality.[1] This paper outlines the proactive approach taken in Canada since 2003. There are only two collectors and distributors of blood products. Héma-Québec for the province of Quebec, and Canadian Blood Services (CBS) for the rest of Canada. This centralized system allows for a uniform plan to be developed for reporting, testing and categorization of all severe transfusion reactions, including TRALI. A reporting requirement for severe adverse transfusion reactions such as TRALI, is stipulated by the Canadian Standards Association (CSA) Standard Z902-04 “Blood and Blood Components". Hospitals report TRALI or possible TRALI reactions to the respective blood supplier, which in turn must report reactions to Health Canada, the federal blood regulator.[2] To formulate a standardized approach to TRALI surveillance for the country, an international consensus meeting was convened.

## Consensus Conference: April 2004

In his introduction to the meeting entitled “Towards an Understanding of TRALI”, the Chairman, Dr. Morris Blajchman of Hamilton, stated that “despite the increasing recognition of TRALI, much about the clinical syndrome remains still poorly understood”. He also proposed that, “It is our hope through this process of scientific investigation followed by open discussion, that the TRALI Consensus Panel will be able to respond effectively to the six questions posed by the Conference Steering Committee … and be able to provide recommendations and guidelines to help in future decision-making for TRALI patients and for donor issues related to TRALI”.[3]

The Conference questions designed ahead of time by the Steering Committee were as follows:

1. The magnitude of the risk of TRALI is unknown at this time. What processes should be implemented to better define the magnitude of the TRALI risk?
2. A variety of respiratory complications can be associated with transfusion. How should TRALI be defined and what processes should be implemented in order to develop objective criteria for use in the classification of TRALI reactions?
3. What are the potential pathophysiologic mechanisms leading to TRALI and what research questions should be explored to investigate the mechanism(s) leading to TRALI?
4. What options are available for managing donors implicated in TRALI reactions?
5. Is there sufficient evidence, at this time, to recommend that any laboratory screening tests and/or other deferral measures be implemented to exclude donors in order to reduce the risk of TRALI?
6. What further information and systematic research is necessary to better evaluate the issues of the epidemiology and pathophysiology of TRALI, in order to reduce the risk to transfusion recipients?

### Consensus Panel Report

The details of the Panel's deliberations can be found in the Review published in TRANFUSION, Dec. 2004 and are summarized below:[4]

### Question 1
Processes to define the magnitude of risk

A true picture of the incidence of TRALI is difficult to ascertain because of disparate definitions and laboratory diagnostic criteria. Outcomes also were unclear, although an incidence of 17% of severe reactions and a mortality rate of between five and ten percent are suggested in the literature. The panel recommended a definition and its universal adoption. In addition, they recommended the enhancement of existing surveillance systems and the establishment of such systems in jurisdictions where they are not present.

### Question 2
The definition of TRALI

The first task was to arrive at a definition of TRALI or possible TRALI and to distinguish these from other causes of acute lung injury (ALI). A “true” TRALI reaction is acute in nature, occurs within six hours of transfusion, and occurs in the absence of an alternative risk for ALI. Hypoxemia and bilateral infiltrates on chest X-ray should be present, and there should be no evidence of circulatory overload. The definition of a “possible” TRALI reaction would satisfy the above criteria but would be associated with alternative risk factors for ALI e.g., sepsis, multiple trauma, severe burn injury, etc. An attempt was made to define the syndrome in a manner that all surveillance systems could document it in a standardized way.

### Question 3
Pathophysiology

It was recognized that there were two proposed pathophysiologic systems leading to TRALI: the antibody hypothesis; and the neutrophil priming hypothesis.[5] Details are beyond the scope of this paper.

### Question 4
The Management of Donors

The panel defined a donor as “associated” if one or more of his or her blood components was transfused during the six hours preceding the first clinical manifestations of TRALI. The donor is defined as “implicated” in TRALI if antibodies to HLA class I or II antigens or antibodies to Human Neutrophil Antigens (anti-HNA-3a) with specificity against an antigen present on the recipient's white cells are detected. Discussions of lab evaluations of donors and acceptability of various blood fractions of these donors was felt to require further consideration and might well depend on availability of blood resources. The management of “associated” and “implicated” donors could entail by either using their washed red blood cells only and discarding plasma containing products or permanent deferral.

### Question 5
Screening: Tests and deferrals

The panel agreed there were no suitable lab screening tests for donors. It therefore focused on proposals to restrict potential “high risk” donors, with a realization of the negative impact this may have on the donor base. These “high risk donors” may include all female donors or only those previously pregnant; all transfused donors; and donors with previously demonstrated WBC antibodies. There are three other strategies involving products that also were discussed, although they were not recommended at the time of the report. These were: the use of fresher blood to avoid priming for the two-event pathophysiologic model; the use of solvent detergent plasma products; and the use of platelet storage solutions in order to dilute the plasma.

### The steps that have been taken by Canadian Blood Services the collection and review of reaction data

A TRALI Review Group was established by Canadian Blood Services to collect and process all reports. The purposes of the TRALI Review Group were to develop national policies, review the results of individual case investigations, and determine future donor management. Héma-Québec has a separate Hemovigilance system, and processes its reports separately. Elsewhere in Canada, all reports from hospitals are directed to the Medical Directors at the regional CBS Centres, who ensure that all clinical documentation is in place. This includes information regarding the time and duration of the transfusion, the time lapsed before the reaction, the type and number of products transfused, blood gas information, chest X-ray reports, and other clinical information. All reports of TRALI or possible TRALI must be reported by the blood supplier (i.e. CBS or Hema-Quebec) to Health Canada within 15 days.[2] Relevant information is documented on a standardized Canadian Transfusion Adverse Event Reporting Form.[6] Any deaths related to a probable or possible TRALI reaction must be reported to Health Canada within 24h. Aggregate, non-nominal, national hemovigilance data on TRALI and other severe transfusion reactions that are reported to Health Canada are periodically summarized and publicly disseminated by the Canadian Transfusion Transmitted Injury Surveillance System (CTTISS).[7] CTTISS is a national surveillance and monitoring system in Canada for reporting of adverse reactions to blood and blood products.

### The Management of Donors Associated with TRALI (Secondary Prevention)

In the case of a suspected TRALI, all donor products are removed from inventory at CBS or in the hospitals and placed in quarantine. If a product has been transfused, the treating hospital is notified to follow-up with the recipient. Donor information is entered into the CBS national computer system but donors may donate plasma only for fractionation with negative TRALI test results, pending the resolution of the case. Pending the results of TRALI-specific blood testing. CBS has developed an algorithm[8] for managing test results for possibly implicated and implicated donors. Samples from the donor are collected and sent to a National Reference Lab for determination of anti-HLA1 and 2 antibodies, as well as anti-HNA-3a antibodies. CBS also endeavours to collect recipient samples for similar testing and cross-matching against donor plasma.

A donor is definitely implicated if shown to have: a positive cross match with the recipient's leukocytes, or, an antibody with specificity corresponding to a known recipient cognate antigen, or, an antibody with HNA-3a specificity. A donor is possibly implicated if shown to have: a leukocyte antibody but is cross match untested with the recipient, or, antibody specificity is not defined or is untested, or, recipient cognate antigen type is not known. All donors who are definitely implicated are permanently deferred and in-date components withdrawn. Washed RBCs may be used if the person is a rare blood type donor. Donors who are possibly implicated may continue to donate red blood cells if they are a rare blood type. These red cells are frozen and/or washed. Their whole plasma can be sent for fractionation. Large volume plasma products such as frozen plasma (FP), cryoprecipitate, and platelets from these donors are discarded. All in-date components are withdrawn.

In regards to primary prevention, CBS has followed the patterns of practice in the UK and Europe as well as the recommendations from the American Association of Blood Banks (AABB).[9] CBS is in the process of implementing measures to institute the use of predominately male plasma[10] in such plasma containing products as FP, FFP, Cryosupernatant Plasma, Apheresis Plasma and liquid plasma for resuspending CPD Pooled Platelets. The majority of plasma from female whole blood donors will be sent to fractionators to be processed into plasma protein products such as intravenous gamma globulin (IVIG) and albumin. In the future, CBS plans to direct all Source Plasma collected from female donors by apheresis for fractionation. Despite the move towards predominantly male plasma for transfusion, plasma from female donors continues to be important to support the needs of Canadian patients and may still be issued to meet group or component specific urgent shortfalls.[11] Planning is currently underway to determine how CBS can implement TRALI reduction guidelines for platelet apheresis units with a target of late 2008. It is hoped that these measures will contribute significantly to TRALI risk reduction.

## Summary and Conclusions

As TRALI is one of the leading causes of transfusion associated mortality in Canada, it is essential that the Canadian Blood Services apply all measures to reduce its risk. CBS has been active in educating its center physicians, staff and the hospital community[12] regarding the definition of TRALI and how to report it. A central TRALI Review Group has been established to assist local Medical Directors in managing reported cases of TRALI in addition to collecting statistics on its incidence and prevalence. Both primary and secondary prevention strategies are in place to mitigate the risk within our donor population and within our inventory. It is essential that the hemovigilance systems continue to track carefully the incidence of this condition in order to verify that the protective measures have had their expected impact.
